# Design of a filtration system to improve the dose distribution of an accelerator‐based neutron capture therapy system

**DOI:** 10.1002/mp.15864

**Published:** 2022-08-19

**Authors:** Naonori Hu, Hiroki Tanaka, Koji Ono

**Affiliations:** ^1^ Kansai BNCT Medical Center Osaka Medical and Pharmaceutical University Osaka Japan; ^2^ Particle Radiation Oncology Research Center Kyoto University Institute for Integrated Radiation and Nuclear Science Osaka Japan; ^3^ Kansai BNCT Joint Clinical Institute Osaka Medical and Pharmaceutical University Takatsuki Osaka 569‐8686 Japan

**Keywords:** boron neutron capture therapy, Monte Carlo simulation, neutron moderator, thermal neutrons

## Abstract

**Purpose:**

The aim of this study is to design and evaluate a neutron filtration system to improve the dose distribution of an accelerator‐based neutron capture therapy system.

**Methods:**

An LiF‐sintered plate composed of 99%‐enriched ^6^Li was utilized to filter out low‐energy neutrons to increase the average neutron energy at the beam exit. A 5‐mm thick filter to fit inside a 12‐cm diameter circular collimator was manufactured, and experimental measurements were performed to measure the thermal neutron flux and gamma‐ray dose rate inside a water phantom. The experimental measurements were compared with the Monte Carlo simulation, particle, and heavy ion transport code system. Following the experimental verification, three filter designs were modeled, and the thermal neutron flux and the biologically weighted dose distribution inside a phantom were simulated. Following the phantom simulation, a dummy patient CT dataset was used to simulate a boron neutron capture therapy (BNCT) irradiation of the brain. A mock tumor located at 4, 6, 8 cm along the central axis and 4‐cm off‐axis was set, and the dose distribution was simulated for a maximum total biologically weighted brain dose of 12.5 Gy with a beam entering from the vertex.

**Results:**

All three filters improved the beam penetration of the accelerator‐based neutron source. Filter design C was found to be the most suitable filter, increasing the advantage depth from 9.1 to 9.9 cm. Compared with the unfiltered beam, the mean weighted dose in the tumor located at a depth of 8 cm along the beam axis was increased by ∼25%, and 34% for the tumor located at a depth of 8 cm and off‐axis by 4 cm.

**Conclusion:**

A neutron filtration system for an accelerator‐based BNCT system was investigated using Monte Carlo simulation. The proposed filter design significantly improved the dose distribution for the treatment of deep targets in the brain.

## 1 INTRODUCTION

Boron neutron capture therapy (BNCT) is a binary treatment modality where a boron compound is administered into the patient, followed by neutron beam irradiation. The nuclear reaction that occurs when a thermal neutron is captured by a ^10^B atom produces high linear energy transfer particles (alpha particle and lithium nucleus) that travel a few micrometers inside the human tissue. Therefore, the dose is given to a small area where this reaction took place, the cells containing the boron compound, minimizing the dose to the surrounding healthy tissue. The world's first clinical BNCT for cancer was performed using a research reactor in the USA in 1951.^1^ The majority of BNCT conducted worldwide used neutrons generated from nuclear reactors. In Japan, at the Kyoto University, Institute for Integrated Radiation and Nuclear Science (KURNS), over 500 clinical irradiations, had been carried out using the Kyoto University Research Reactor (KUR) between 1990 and 2014.^2^, ^3^, ^4^, ^5^

Nowadays, the emerging trend is to consider accelerator‐based neutron systems as a therapeutic option because such systems have several proven advantages over nuclear reactors from a clinical facility perspective. The world's first accelerator‐based BNCT system used in clinical trials was designed and developed by Sumitomo Heavy Industries in collaboration with Kyoto University in 2008.^6^ It is a cyclotron‐based system that utilizes a 30‐MeV proton beam with a beryllium target to generate fast neutrons. The fast neutrons traverse a moderator that slows the neutrons down to the epithermal energy range suitable for BNCT.

**FIGURE 1:**
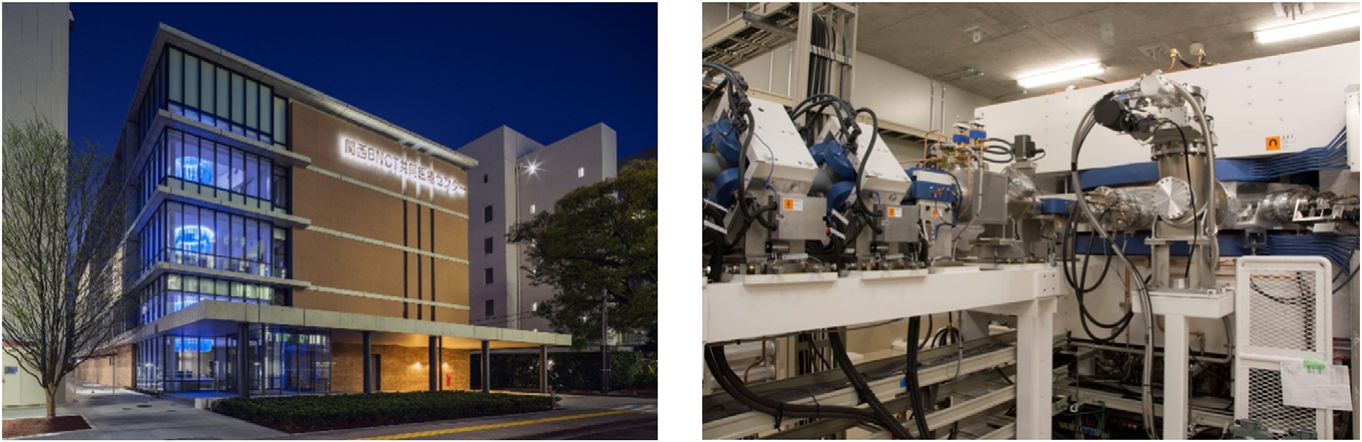
Left: The Kansai boron neutron capture therapy (BNCT) Medical Center. Right: The accelerator‐based BNCT system (NeuCure). Image courtesy of Sumitomo Heavy Industries, Ltd.

Between February 2016 and June 2018, a multi‐institutional open‐label, phase II clinical trial of BNCT with borofalan, which is ^10^B‐labeled boronophenylalanine (BPA), for recurrent malignant gliomas was conducted using the previously mentioned accelerator‐based BNCT system.^7^ The same type of accelerator was installed at the Kansai BNCT Medical Center in 2018 (Figure 1). The center opened in June 2018 to perform both BNCT and PET imaging services. It is located directly next to the Osaka Medical and Pharmaceutical University Hospital, so patients can receive BNCT treatment at the center and return to the hospital for recovery and discharge. This is the world's first accelerator‐based BNCT center situated on university grounds that provides both clinical BNCT services and performs ongoing research and education on BNCT. Following the results of a clinical trial for recurrent head‐and‐neck cancer conducted in parallel with a clinical trial for recurrent malignant gliomas, this accelerator system received medical device approval from the Japanese Ministry of Health, Labor and Welfare in March 2020 (NeuCure) and in June 2020, the system was approved for reimbursement by the national health insurance system for unresectable, locally advanced, and recurrent carcinoma of the head‐and‐neck region. The first head‐and‐neck patient at this center (covered under the national insurance system) was treated on 17 June 2020.

Tanaka^6^ compared the neutron spectrum of the cyclotron‐based neutron source with the KUR epithermal neutron source. Despite the accelerator system having a more penetrating beam than the KUR epithermal neutron beam, the maximum treatable depth is ∼7–8 cm, assuming the boron concentration in the tumor relative to the surrounding brain is approximately three times higher. For tumors that are located deeper than 7–8 cm, the dose limit of the healthy brain governs the curable dose level at the tumor region.

^6^Li is widely used to shield low‐energy neutrons. It has a nuclear cross section that is inversely proportional to the speed of the neutron, with its shielding effect becoming larger for the lower energy neutrons. ^6^Li is preferred over ^10^B in shielding thermal neutrons as it produces little undesired secondary radiation. An ^6^Li‐enriched filter to absorb the low‐energy neutrons for a reactor‐based BNCT system has been performed by other authors.^8^, ^9^, ^10^ This study aims to implement an enriched LiF filter for an accelerator‐based BNCT system to further improve the beam penetration to treat deep‐seated tumors.

## 2 MATERIALS AND METHODS

### 2.1 Monte Carlo simulation to design an LiF filter

**FIGURE 2:**
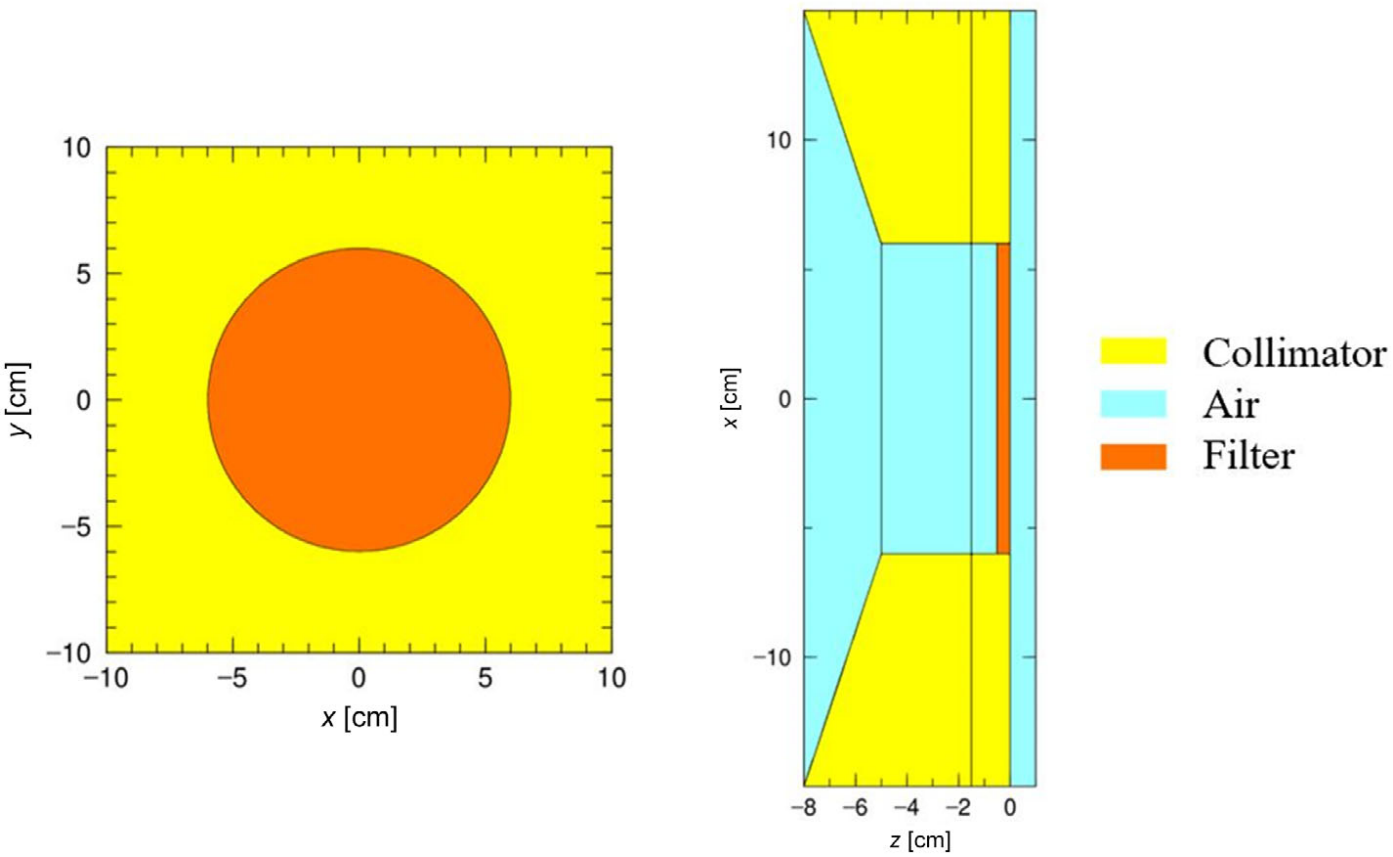
Geometry of design a filter modeled in particle and heavy ion transport code system (PHITS). Left: Beams eye view. Right: Cross‐sectional view with the neutron beam generated from left to right. The yellow region represents the collimator (polyethylene loaded with natural LiF), light blue region represents air, and the orange region represents the filter (LiF).

A Monte Carlo simulation package called particle and heavy ion transport code system (PHITS) was used in this study. Neutron‐induced nuclear reactions were simulated using Japanese Evaluated Nuclear Data Library (JENDL‐4.0).^11^ The target and the beam shaping assembly (BSA), which is composed of lead, iron, aluminum, and calcium fluoride (shown in detail in a separate publication by Tanaka et al.^12^), were modeled, and the neutron and gamma‐ray spectra generated from the target were simulated. Unfortunately, the neutron and gamma‐ray spectra cannot be made available to the reader due to commercial reasons. To reduce the simulation time for subsequent simulations, the neutron and gamma‐ray spectra exiting from the BSA (the lead surface, *z* = −8 cm shown in Figure 2) were tallied, and a circular planar source with a radius of 150 cm (taking into account the angular distribution) was set. A filter made from LiF (enriched with 99% ^6^Li) was modeled to entirely cover the beam exit of a 12‐cm diameter circular collimator (orange region in Figure 2, henceforth known as filter design A). The collimator (yellow region in Figure 2) was composed of polyethylene loaded with natural LiF (mass ratio 50/50). The simulations were executed until the relative standard error at a depth of 15 cm along the central beam axis was less than 1% (∼10^10^ particles were simulated). The neutron and gamma‐ray distribution were tallied with a bin size of 2 mm in all directions. The thermal neutron flux along the central beam axis inside a 20 cm × 20 cm × 20 cm water phantom was simulated. The thickness of the filter was varied from 2.5 to 20 mm with an increment size of 2.5 mm. Along with the thermal neutron distribution, the absorbed dose rate inside the same phantom (material set to ICRP brain^13^) was simulated. The four main dose components (boron, nitrogen, hydrogen, and gamma‐ray dose rate) were evaluated individually using KERMA approximation.

Assuming that the water in the phantom was a healthy brain, and the tumor was present in it, the dose was calculated at the ^10^B concentration of 25 µg g^−1^ and the concentration in the tumor being 3.5 times higher than that of healthy brain.^14^ The relative biological effectiveness for nitrogen, hydrogen, and gamma rays were assumed to be 3.0, 3.0, and 1.0, respectively. The compound biological effectiveness (CBE) values for tumor, skin, and healthy tissue were assumed to be 3.8,^15^ 2.5,^16^ and 1.34,^17^ respectively. The tumor to blood ^10^B ratio and skin to blood ^10^B ratio was assumed to be 3.5 and 1.0, respectively. Details on the BNCT dose component and calculation are shown in the appendix. To evaluate the performance of a neutron beam for neutron capture therapy, a parameter called advantage depth (AD) is commonly used.^18^ AD is defined as the depth where the biologically equivalent dose (Gy) in the tumor region equals the peak value of the biologically weighted dose in the healthy tissue region. A maximum total biologically equivalent dose of 12.5 Gy for brain was set. This value was adopted from the study performed by Morris.^19^ Along with the AD, the treatment time to deliver 12.5 Gy to the brain was calculated (maximum treatment time: MTT).

### 2.2 Filter manufacturing and experimental measurement

**FIGURE 3:**
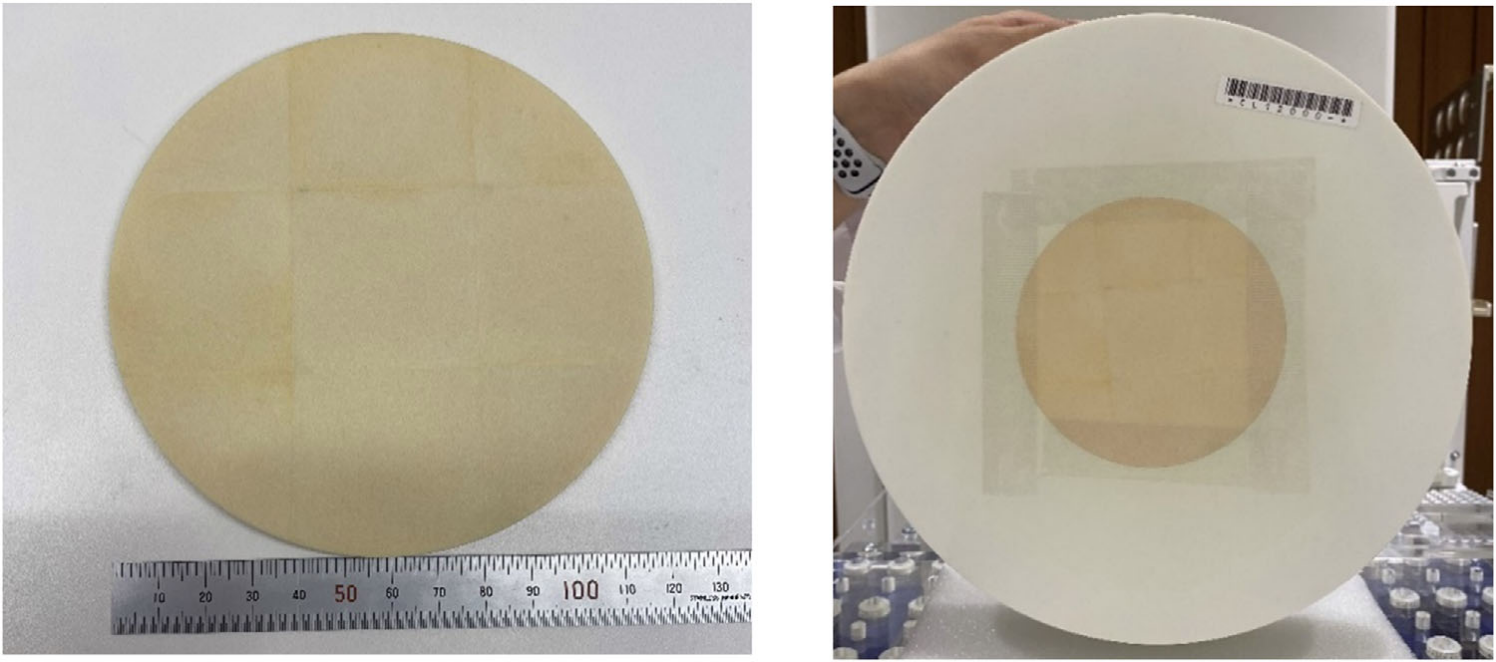
Image of the manufactured 5‐mm‐thick LiF filter placed inside the 12‐cm diameter collimator

Based on the results of the Monte Carlo simulation, the filter material was made up of a mixture of enriched ^6^LiF and natural LiF (mass ratio of ∼60/40). The ^6^LiF powder, which was composed of 99% enriched ^6^Li, was purchased from Sigma‐Aldrich, and the manufacturing process was performed by Nikkei Sangyo Co., Ltd., Nippon Light Metal Group. Nine pieces of a square sintered plate (*W*41 mm × *H*41 mm × *t*2.5 mm) were put together using an adhesive bond (40% ^6^LiF powder, 60% epoxy resin). Two layers were produced to create a single plate (*W*123 mm × *H*123 mm × *t*5 mm). The square plate was machined to fit a circular collimator having a diameter of 120 mm (Figure 3).

To validate the simulation results, experimental measurement was performed using a PMMA phantom filled with distilled water. A thin gold wire (diameter of 0.25 mm) was placed along the central beam axis with and without a cadmium cover in the water phantom to determine the thermal neutron flux. Off‐axis thermal neutron flux was also measured at a depth of 2, 4, and 6 cm inside the water phantom.

After neutron irradiation, the gamma rays emitted from the activated gold sample was measured using a high‐purity germanium detector (ORTEC ICS‐P4). The reaction rate of the gold sample was calculated using the following expression:

$$R=\frac{\lambda C}{\varepsilon \gamma e^{-\lambda T_{C}}\left(1-e^{-\lambda T_{m}}\right)\sum_{i=1}^{n}\left(\frac{Q_{i}}{\Delta t}\left(1-e^{-\lambda \Delta t}\right)e^{-\lambda \left(n-i\right)\Delta t}\right)}$$

where ε is the detection efficiency of the detector of the gamma rays emitted from ^198^Au, *ɣ* is the gamma‐ray emission rate from ^198^Au decay, *λ* is the decay constant of ^198^Au, *T_C_* is the time from the irradiation to the start of the measurement, *T_m_* is the measurement time, *C* is the peak count due to the detector measured gamma rays emitted from ^198^Au, and *Q_i_* is the electric charge irradiated on the target at each interval, Δ*t*.

**FIGURE 4:**
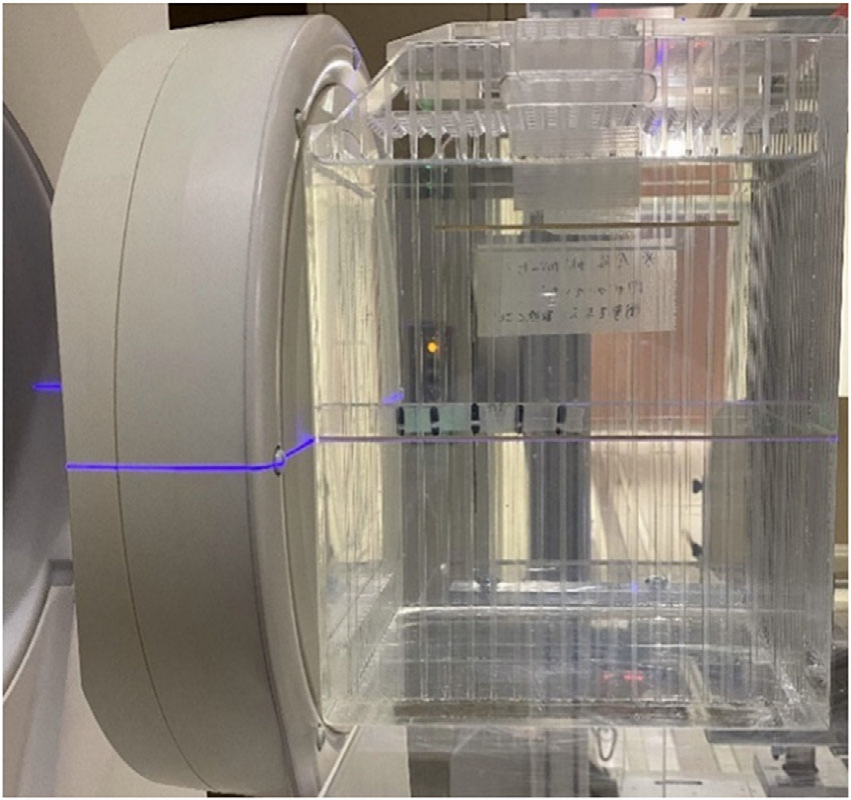
Experimental setup showing the water phantom with thermo‐luminescent dosimeters (TLDs) placed along the beam axis

Thermo‐luminescent dosimeters (TLDs) were used for the measurement of the gamma‐ray dose rate along the beam axis (Figure 4). Commercially available BeO powder TLD is usually encapsulated in borosilicate glass, which has a high sensitivity to thermal neutrons. Therefore, a special‐ordered BeO TLD enclosed in a quartz glass capsule was used to measure the gamma‐ray dose rate in the phantom. This TLD has been used previously by Sakurai et al. at the KUR^20^ and was calibrated using a Co‐60 source.

### 2.3 Simulation study to investigate a more optimum filter

**FIGURE 5:**
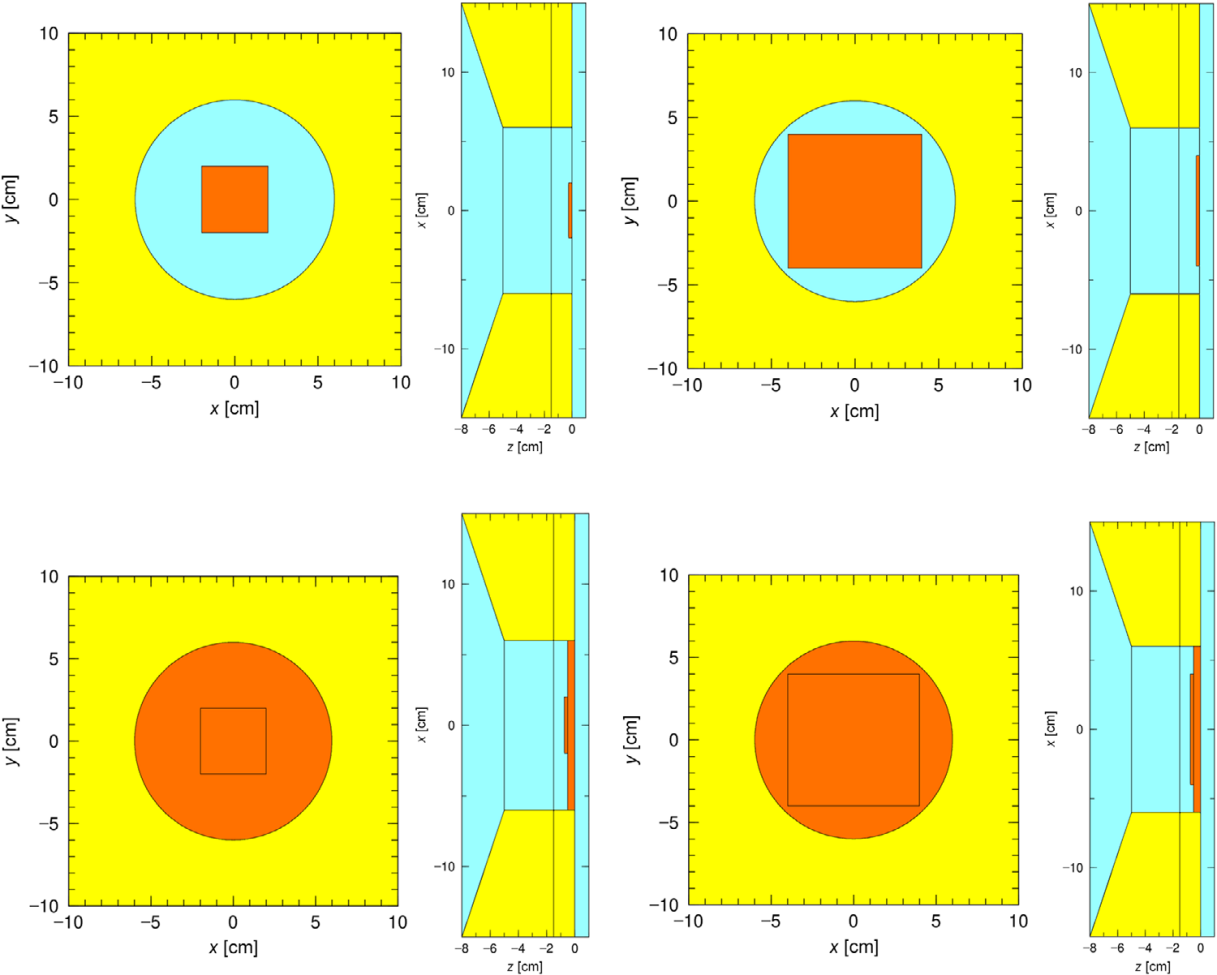
Top: Geometry of design B filter 4 cm × 4 cm (left) and 8 cm × 8 cm (right) modeled in particle and heavy ion transport code system (PHITS). Bottom: Geometry of design C with 4 cm × 4 cm (left) and 8 cm × 8 cm (right) filter modeled in PHITS

**FIGURE 6:**
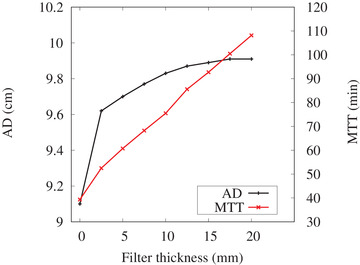
The advantage depth (AD) and maximum treatment time (MTT) as a function of filter thickness for design A

Following the validation of the Monte Carlo simulation, two other filter configurations were investigated to further optimize the dose distribution:

1. Design B: Uniform thick 4 cm × 4 cm and an 8 cm × 8 cm square plate (partially covering the beam exit) placed at the center of the field (Figure 5).
2. Design C: The combination of the manufactured 5‐mm thick 12‐cm diameter uniform filter and the previous square plate placed at the center (Figure 5). (Figure 6)

### 2.4 Thermal neutron distribution and biologically weighted dose distribution

The thermal neutron flux along the central beam axis was simulated inside a 20 cm × 20 cm × 20 cm water phantom for the previous filter designs. The biologically weighted dose distribution along the beam axis and off‐axis at a depth of 2 cm was simulated for the different designs. The neutron and gamma‐ray flux inside the water phantom was scored using the T‐track tally with an *xyz* geometric mesh with a uniform mesh size of 0.5 mm. The T‐track tally is used to obtain the fluence in any specified region. The track length is evaluated when a particle passes through the region, and the sum of the track length is scored, and the particle fluence, units of cm^−2^ per source, is determined by dividing the scored track lengths by the volume of the region and the total number of source particles.

### 2.5 3D dose distribution inside using a dummy patient dataset

A CT dataset of a patient used for training and education (provided by RaySearch Laboratories) was used, and the images were converted into a voxel phantom using a PHITS2DICOM module. The voxel size was set to a 2‐mm cube, and the conversion of CT number to material density was estimated using the data published by Schneider.^21^ A 2‐cm diameter spherical mock tumor was placed at a depth of 4 cm (Tumor 1), 6 cm (Tumor 2), 8 cm (Tumor 3) along the beam axis and at a depth of 8 cm located 4‐cm off‐axis (Tumor 4). A 12‐cm diameter beam entering from the vertex was placed, and the dose distribution was simulated. The 3D dose distribution was imported into an open‐source software called Slicer,^22^ and the dose–volume histograms of the tumors, brain, and the body region were analyzed.

## 3 RESULTS

### 3.1 Monte Carlo simulation to design and manufacture a LiF filter

The Monte Carlo simulation result showed both the AD and MTT increased with increasing filter thickness. The AD increased by several millimeters with a filter thickness of 2.5 mm, slowly increased with added thickness, and eventually saturated at a thickness of ∼17.5 mm, shown in Figure 6. To keep the MTT ∼60 min, a filter thickness of 5 mm was chosen and manufactured.

**FIGURE 7:**
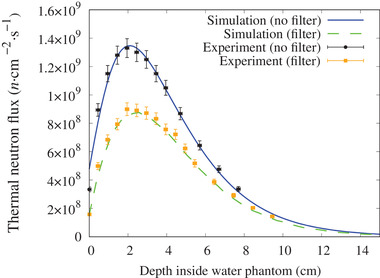
Thermal neutron flux along the central beam axis inside the water phantom with (dash line) and without (solid line) the LiF filter for a 12‐cm diameter circular field

**FIGURE 8:**
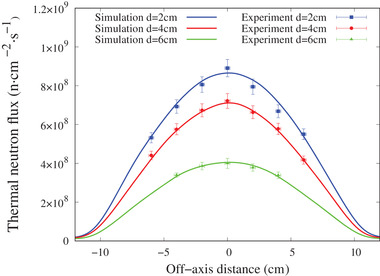
Off‐axis thermal neutron flux at a depth of 2, 4, and 6 cm inside the water phantom with the LiF in place for a 12‐cm diameter circular field

**FIGURE 9:**
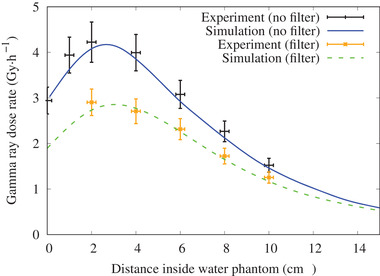
Gamma‐ray dose rate along the beam axis inside the water phantom with (dash line) and without (solid line) the LiF filter for a 12‐cm diameter circular field

The experimental and simulated thermal neutron flux along the central axis and off‐axis with and without the 5‐mm thick ^6^LiF filter are shown in Figures 7 and 8, respectively. The gamma‐ray dose rate along the beam axis is shown in Figure 9. The simulation results closely matched the experimental measurement, within the experimental uncertainty.

### 3.2 Simulation of biologically weighted dose distribution for the various LiF filter designs

The thermal neutron flux and the biologically weighted dose distribution for each individual dose component along the central axis for the three filter designs with various thicknesses are shown in the appendix.

**FIGURE 10:**
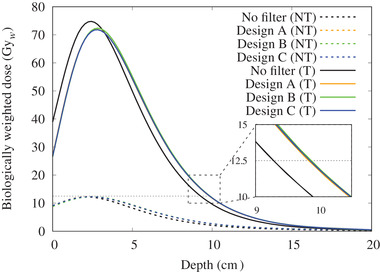
The biologically weighted dose rate along the central axis simulated inside a tissue phantom for the different filter designs. The solid lines indicate the tumor dose distribution (*T*) and the dashed dotted lines indicate the normal tissue (NT) dose distribution

**FIGURE 11:**
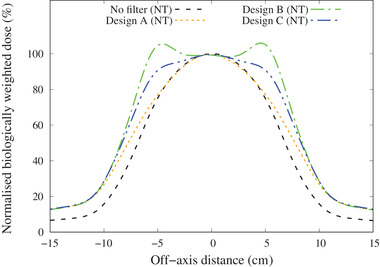
The off‐axis biologically weighted dose rate at a depth of 2 cm for the different filter designs (normalized at the center)

**FIGURE 12:**
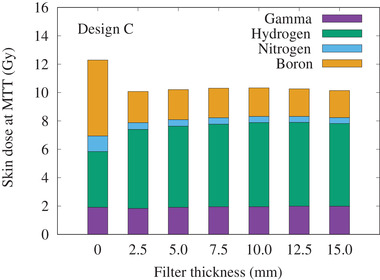
The skin dose (separated into each dose component, biologically weighted dose) for filter design C when the maximum total biologically weighted dose of the brain was 12.5 Gy

The total biologically weighted dose rates for normal tissue (NT) and tumor (*T*) for the unfiltered beam and for the three filters (design A: *∅*120 mm × *t*10 mm, design B: *W*80 mm × *H*80 mm × *t*10 mm, design C: *∅*120 mm × *t*5 mm + *W*80 mm × *H*80 mm × *t*5 mm) are shown in Figure 10. The off‐axis total biologically weighted dose distribution at a depth of 2 cm is shown in Figure 11. The relationships between AD and MTT are shown in the appendix and summarized in Table 1. All three filters increased the AD by almost exactly the same amount. The MTT was the shortest with design B; however, the off‐axis dose profile indicated an increase in dose at the edge of the field. In contrast, design C produced a flatter beam with a similar MTT to design A. Figure 12 shows the change in skin dose as the filter thickness was increased for a maximum total biologically weighted dose of 12.5 Gy delivered to the brain for filter design C.

**TABLE 1:** The advantage depth (AD) and maximum treatment time (MTT) for the different filter designs

Filter design	AD (cm)	MTT (min)	Skin dose (Gy)
No filter	9.1	39.4	12.3
Design A	9.8	75.5	10.5
Design B	9.9	69.7	9.7
Design C	9.9	75.6	10.2

### 3.3 3D dose distribution inside a sample patient dataset

**FIGURE 13:**
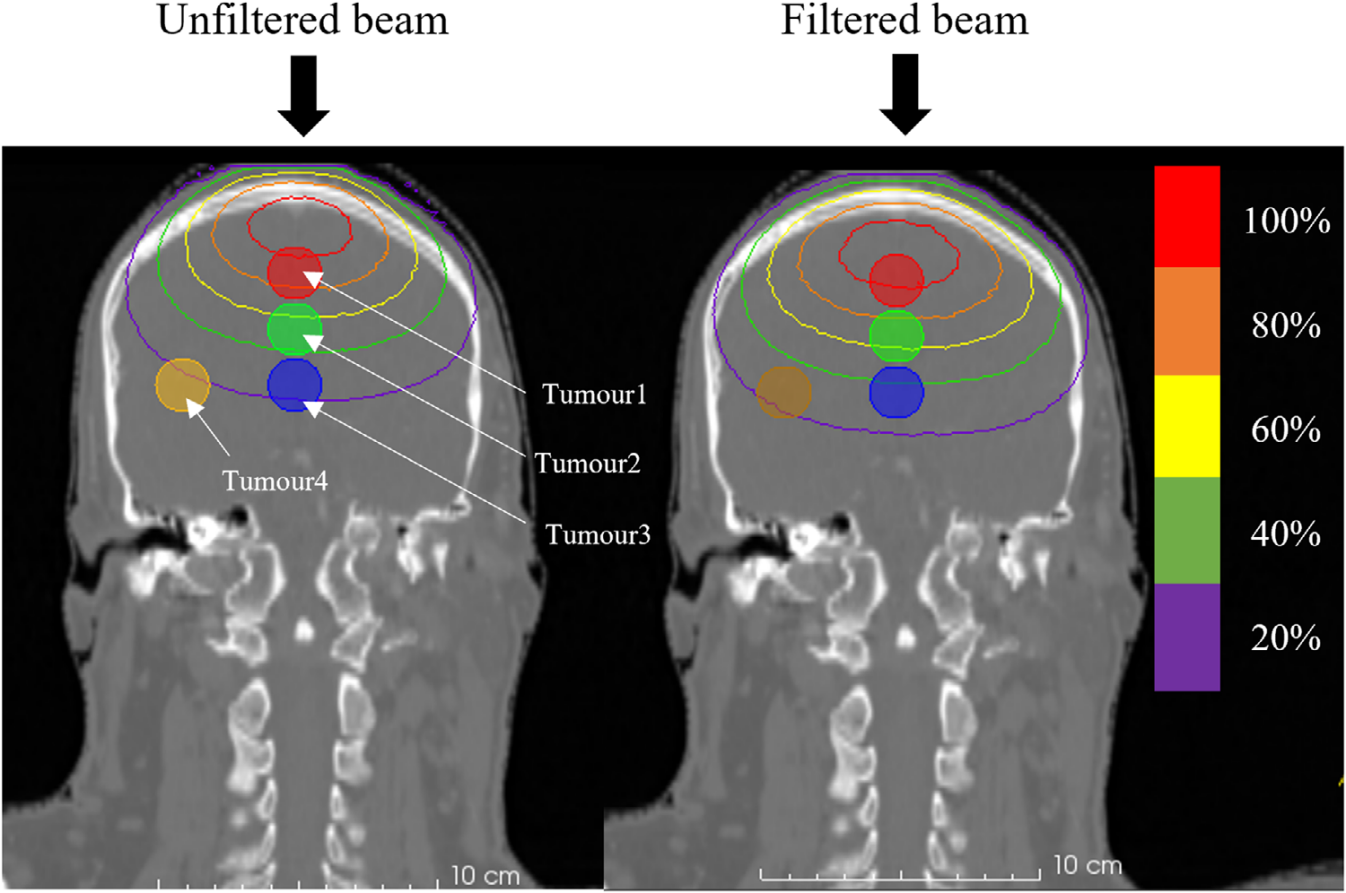
Isodose distribution of the total biologically weighted tumor dose for the unfiltered beam (left) and beam with filter design C (right)

**FIGURE 14:**
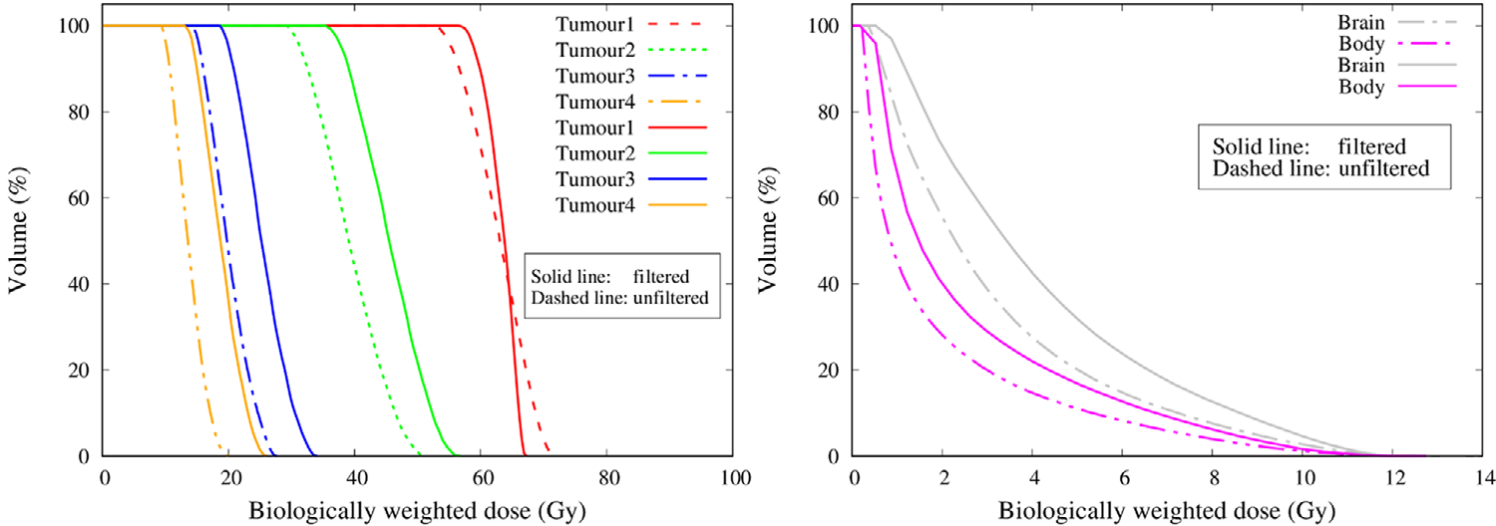
Dose–volume histograms of the four tumors (left) and the brain and body region (right). The dashed lines indicate the unfiltered beam and the solid line indicates the beam with the filter design C

The isodose distribution of the total biologically weighted dose with and without the filter design C is shown in Figure 13. The tumor dose distribution in the depth and lateral direction was improved with the filter in place. The DVH of the tumors and the NT and the corresponding parameters are shown in Figure 14 and Table 2, respectively. The DVH comparison between the unfiltered beam and the beam with filter design C showed that the tumor dose distribution was improved with the filtered beam, both along the beam axis and off‐axis, by up to 34%.

**TABLE 2:** The biologically weighted dose–volume histogram parameters of the tumors, brain, and the body region for the sample patient

Structure	Biologically weighted dose (Gy)								HI (*D*_5_/*D*_95_)	
	*D*_5_		*D*_98_		*D*_50_		*D*_95_			
	No filter	Filter	No filter	Filter	No filter	Filter	No filter	Filter	No filter	Filter
Tumor 1	69.7	66.5	54.2	58.1	63.1	63.8	55.4	58.9	1.3	1.1
Tumor 2	48.0	53.6	30.4	36.9	39.1	45.5	31.3	37.9	1.5	1.4
Tumor 3	25.7	31.8	14.9	19.4	19.8	25.3	15.4	20.0	1.7	1.6
Tumor 4	18.0	24.0	9.8	13.9	13.6	18.8	10.2	14.4	1.8	1.7
Brain	0.3	0.5	12.5	12.5	2.3	3.4	0.6	1.0	–	–
Body	0.0	0.0	13.6	12.5	0.8	1.5	0.3	0.5	–	–

Abbreviation: HI, homogeneity index.^23^

Using the equation defined by Wang et al.^23^ (homogeneity index = *D*_5_/*D*_95_), the homogeneity index (which indicates the degree of uniformity of dose distribution inside the target volume) for the tumors decreased by a ∼5%–15% with LiF filter (design C).

## 4 DISCUSSION

The neutrons exiting from the BSA for BNCT has a broad energy spectrum, including low‐energy neutrons that are troublesome for the treatment of deep‐seated tumors. This is because these low‐energy neutrons are captured at the shallow region, increasing the dose delivered to the healthy tissue, which limits the dose being delivered to a deep‐seated tumor. Therefore, by eliminating these low energy neutrons at the beam exit, the dose to the healthy tissue will be reduced and subsequently the dose delivered to the tumor at the deeper region can be increased by increasing the irradiation time.

To eliminate these low‐energy neutrons, a thin LiF filter was placed at the beam exit. The LiF was selected due to the high nuclear cross section at low neutron energies, high chemical stability, and the production of very little undesired secondary radiation. The simulation results indicated that as the thickness of the filter was increased, the thermal neutron flux inside the water phantom at the shallow region decreased, which resulted in an increase in the beam penetration parameter (i.e., AD). However, as the filter thickness increased, the MTT also increased. A 20‐mm thick filter would require a long irradiation time to obtain the thermal neutron fluence required for BNCT, making it impractical to apply to the current system. Therefore, based on the simulation results, a 5‐mm thick LiF filter was selected, manufactured, and the experimental measurements confirmed the simulation results. Upon confirming the simulation results, two other designs (B and C) were tested, and the thermal neutron flux and the biologically weighted dose along the central beam axis were simulated. Similar results to design A were obtained (i.e., increasing the filter thickness increased the AD and MTT). The dominant tumor dose component for all filters was the boron dose, resulting in ∼>90% of the total dose. However, for the NT dose the hydrogen dose (dependent on the fast neutron distribution) was found to be the highest at the surface. The dose at the surface (i.e., skin dose) for filter design C showed that the fast neutron component was relatively high. However, with the LiF filter in place, the thermal neutron flux at the surface was significantly reduced (∼50%), resulting in a reduction in both the boron and nitrogen dose component. For a maximum biologically equivalent dose of 12.5 Gy to the healthy brain, the skin dose decreased with the filter in place. In a study performed by Menéndez et al., concluded for patients that received BNCT with BPA, the acceptable toxicity of the skin was 24 Gy.^24^ The simulation results indicated the skin dose to be smaller than this value and the dose limiting organ (for brain BNCT using this system) would most likely be the healthy brain.

This is the world's first application of an ^6^Li‐enriched LiF filter to an accelerator‐based BNCT system to improve the dose distribution of a neutron beam produced by an accelerator (cyclotron). The study performed by Sakurai and Ono^10^ used an ^6^Li filter (for reactor‐based BNCT) covering only the central part of the field (similar to design B). They intended to select a partial shield because if the entire field was covered, the neutron intensity would significantly decrease, and the total treatment time would have been impractical. Even though the partial filter technique improved the treatable depth, the dose at the edge of the field increased (similar result was achieved with design B). In this study, an additional layer of LiF filter covering the entire beam exit was added (i.e., design C), which resulted in the reduction of the horns at the edge of the field and a more uniform flat beam being produced. The use of this nonuniform filter may be useful for the treatment of a wide target. However, the additional layer increased the MTT to 75.6 min, so to apply this filter for clinical use, the neutron intensity will need to be increased to keep the MTT within 1 h. The NeuCure BNCT accelerator system is currently operating with a proton current of 1 mA, with the capacity to operate at 2 mA. Therefore, the increase in the treatment time with the addition of the proposed filter can be compensated by increasing the proton current. So, if the accelerator was to be operated at 2 mA, the MTT for the proposed filter will be ∼37.8 min (50.4 min if operated at 1.5 mA).

According to the recent report of BNCT study for recurrent malignant glioma using an accelerator (same type of accelerator to the one used in this study), the dose prescription for the study was 8.5 Gy to the scalp.^7^ By applying the same dose prescription, the maximum dose delivered to the brain was calculated to be 8.8 Gy, and the dose delivered to a tumor located greater than 8‐cm depth was ∼12.7 Gy without using the filter. Using filter design C and by setting the maximum brain dose to 8.8 Gy, the dose delivered to the tumor at 8‐cm depth was ∼18.2 Gy, an improvement of ∼34%. The report concluded that the accelerator‐based BNCT was safe for patients with recurrent malignant gliomas. The use of this new filter may potentially increase the therapeutic effect, especially for deep‐seated tumors. However, the proposed filter would not be suitable for shallow tumors existing near the surface of the patient. Another type of filter with a different material would be required to shift the dose distribution to the surface, which has been investigated by the author in a separate publication.^25^

As mentioned earlier, insurance covered clinical BNCT is approved for head‐and‐neck cancer in Japan. For the majority of cases, the dose‐limiting organ for head‐and‐neck BNCT is the oral mucosal tissue. Coderre et al. showed the CBE factor for the oral mucosa of a rat using BPA was 4.9,^26^ indicating high sensitivity to boron neutron capture irradiation. This filter reduces the thermal neutron flux at the shallow region, while maintaining a relatively high thermal neutron flux at the deep region. By reducing the thermal neutron fluence delivered to the oral mucosa, the tumor dose at the deeper region will be improved. This raises the potential of this filter to be used to shield the oral mucosa, shaping the dose distribution to deliver a more optimal and patient specific treatment plan. The proposed filter may exhibit more usefulness to head‐and‐neck BNCT, as the CBE factor for oral mucosa is much higher than the healthy brain.

Another method to improve the dose distribution is to use a multifield irradiation technique, which is a method frequently used in X‐ray therapy. However, from a practical and financial point of view, a rotating gantry for BNCT may be difficult. Moreover, for BNCT, it is favorable to keep the distance between the surface of the patient and the beam exit port as short as possible to reduce the treatment time. Having multiple treatment fields would require the patient to be repositioned among fields. The medically approved BPA produced by STELLA PHARMA is only approved for infusion for a total of 3 h (the infusion rate for the first 2 h is 200 mg kg^−1^ h^−1^, and the last hour is 100 mg kg^−1^ h^−1^). The neutron irradiation is performed when the infusion rate is dropped to 100 mg kg^−1^ h^−1^, so the maximum irradiation time (while the BPA is being infused) is limited to 1 h. Considering the time restriction placed on the BPA infusion time, a method to improve the dose distribution using a single irradiation field is indispensable. Currently, development is underway to increase the proton current, while maintaining a constant beam output. So, in the future, the application of this proposed filter along with the increase in the proton beam current will open doors for a new and significantly improved way of BNCT.

## 5 CONCLUSION

The proposed filtration system for an accelerator‐based BNCT system was investigated, and Monte Carlo simulation showed improvement in the beam penetration by using an enriched ^6^Li LiF filter. The filter was manufactured, and experimental measurements confirmed the simulation results inside a water phantom. The accelerator‐based BNCT system with the proposed filter design (a 5‐mm thick disc covering the entire beam exit with added square plate in the center) improved the dose distribution by approximately up to 25% for a tumor located at a depth of 8 cm along the beam axis, and ∼34% for a tumor located at a depth of 8 cm and off‐axis by 4 cm. The use of this filtration system may increase the indication of accelerator‐based BNCT for the treatment of the brain.

## CONFLICT OF INTEREST

The authors have no relevant conflicts of interest to disclose.

## Figures and Tables

**FIGURE 15:**
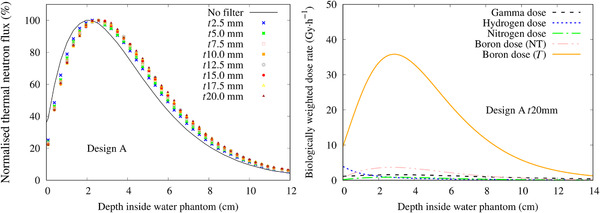
The thermal neutron flux (left) and the biologically weighted dose distribution (right) along the central beam axis for filter design A

**FIGURE 16:**
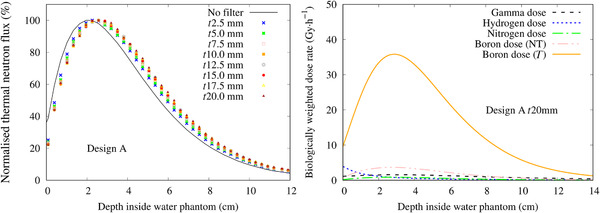
The thermal neutron flux (left) and the biologically weighted dose distribution (right) along the central beam axis for filter design B (4 cm × 4 cm square plate)

**FIGURE 17:**
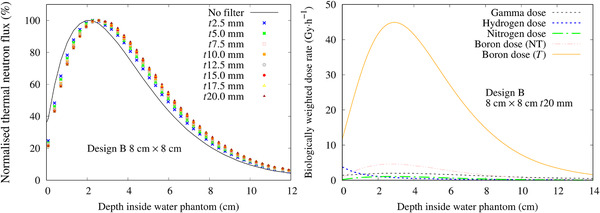
The thermal neutron flux (left) and the biologically weighted dose distribution (right) along the central beam axis for filter design B (8 cm × 8 cm square plate)

**FIGURE 18:**
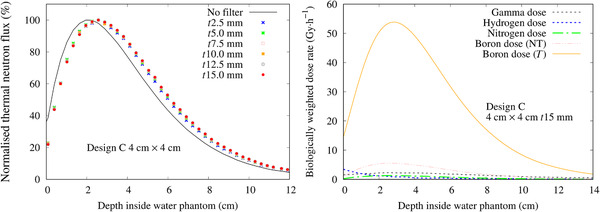
The thermal neutron flux (left) and the biologically weighted dose distribution (right) along the central beam axis for filter design C with 4 cm × 4 cm square plate

**FIGURE 19:**
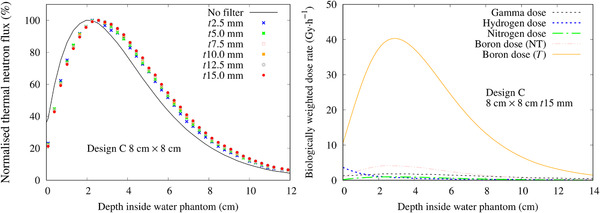
The thermal neutron flux (left) and the biologically weighted dose distribution (right) along the central beam axis for filter design C with 8 cm × 8 cm square plate

**FIGURE 20:**
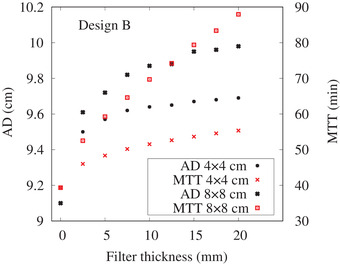
The advantage depth (AD) and maximum treatment time (MTT) as a function of filter thickness for filter design B

**FIGURE 21:**
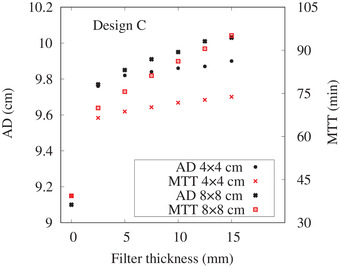
The advantage depth (AD) and maximum treatment time (MTT) as a function of filter thickness for filter design C

## BNCT dose component

The total dose delivered to a patient receiving boron neutron capture therapy is defined as the unweighted sum of all dose components, and the sum of the dose components, each weighted by the corresponding compound biological effectiveness (CBE)/relative biological effectiveness (RBE), is known as the biologically weighted dose:

$$D_{w}=CBE\times D_{B}\;+\;RBE_{p}\times D_{p}\;+\;RBE_{n}\times D_{n}\;+\;RBE_{\gamma }\times D_{\gamma }$$

where *D*_w_ represents the total biologically weighted dose, and each dose component is described later.

The boron dose (*D*_B_) is dose due to the ^10^B fission reaction, ^10^B (n, α)^7^Li. The emitted alpha particle and the recoiling ^7^Li ion result in locally deposited energy averaging about 2.33 MeV. The nitrogen dose (*D*_p_) is the dose due to the ^14^N in tissue capturing a thermal neutron and emitting a proton in an ^14^N (n, p)^14^C reaction. The neutron dose (*D*_n_) is the dose due to the epithermal and fast neutrons causing recoil protons from hydrogen in tissue. The gamma‐ray dose (*D*_ɣ_) is the dose due to the gamma rays accompanying the neutron beam as well as gamma rays induced in the tissue itself. *RBE*_p_, *RBE*_n,_ and *RBE*_ɣ_ are *RBE* values of nitrogen, neutron, and gamma rays, respectively. CBE considers the boron distribution in a cell and is dependent on the boron compound type and the type of tissue being evaluated.

## Thermal neutron flux and biologically weighted dose rate along the central beam axis

Figures 15, 16, 17, 18, 19 show the simulated thermal neutron flux and the corresponding biologically weighted dose distribution along the central beam axis for the different filter designs. Figures 20 and 21 show the relationship between the advantage depth and maximum treatment time for the different filter designs.
